# Speaking the Self: How Native-Language Psychotherapy Enables Change in Refugees: A Person-Centered Perspective

**DOI:** 10.3390/healthcare13151920

**Published:** 2025-08-06

**Authors:** Viktoriya Zipper-Weber

**Affiliations:** Department of Economy and Health, University for Continuing Education Krems, 3500 Krems, Austria; viktoriya.zipper-weber@donau-uni.ac.at

**Keywords:** native-language psychotherapy, mother tongue counseling, flight, war, trauma, person-centered

## Abstract

Background: Since the outbreak of war in Ukraine, countless forcibly displaced individuals facing not only material loss, but also deep psychological distress, have sought refuge across Europe. For those traumatized by war, the absence of a shared language in therapy can hinder healing and exacerbate suffering. While cultural diversity in psychotherapy has gained recognition, the role of native-language communication—especially from a person-centered perspective—remains underexplored. Methods: This narrative review with a thematic analysis examines whether and how psychotherapy in the mother tongue facilitates access to therapy and enhances therapeutic efficacy. Four inter-related clusters emerged: (1) the psychosocial context of trauma and displacement; (2) language as a structural gatekeeper to care (RQ1); (3) native-language therapy as a mechanism of change (RQ2); (4) potential risks such as over-identification or therapeutic mismatch (RQ2). Results: The findings suggest that native-language therapy can support the symbolic integration of trauma and foster the core conditions for healing. The implications for multilingual therapy formats, training in interpreter-mediated settings, and future research designs—including longitudinal, transnational studies—are discussed. Conclusions: In light of the current crises, language is not just a tool for access to therapy, but a pathway to psychological healing.

## 1. Introduction

### 1.1. Mental Health of Refugees as a Societally Relevant Issue

The mental health of refugees represents an urgent social concern. Numerous studies have shown that refugees are disproportionately affected by mental disorders such as post-traumatic stress disorder (PTSD) and depression [1,2]. A meta-analysis found clinically relevant symptoms of PTSD (≈31%) [2]. and depression (≈31%) in around one-third of refugees—significantly higher rates than in general populations. Refugees displaced by war in Ukraine since 2022 are particularly burdened: one study reported acute stress disorder in over 90% of surveyed Ukrainians during the first few weeks after their flight [3]. International health organizations therefore emphasize that the psychological and psychosocial needs of refugees must be considered as integral components of humanitarian aid [1]. Neglecting mental health not only impairs individual well-being, but also social integration and the resilience of host communities. Thus, there is a moral imperative to provide refugees with appropriate psychosocial support [1]. The high prevalence of mental disorders and their consequences underscore the overarching societal relevance of this issue. Several authors argue that early and sustained psychosocial support may be beneficial in preventing long-term consequences and reducing social and economic costs associated with untreated trauma [2].

### 1.2. Therapy Language and Native-Language Psychotherapy for Traumatized Refugees

The literature has identified the language in which trauma therapy is conducted as a potentially important factor influencing therapeutic outcomes. Language barriers impede access to psychotherapeutic help and can significantly disrupt emotional and relational work [4]. Several studies have suggested that therapy may be more effective when the therapist and client share a common language, as this can facilitate mutual understanding and emotional expression [5]. One meta-analysis found that therapies conducted in the client’s mother tongue were, on average, twice as effective as those conducted in English for clients with a different first language [5]. Therapy conducted in the client’s native language has been reported to enable more nuanced emotional expression and may help build trust more readily [6]. The use of one’s mother tongue is often associated with feelings of familiarity and psychological safety, which can be particularly relevant in the context of trauma and forced displacement [6]. A shared language may support the development of a therapeutic alliance by enabling clients to express complex emotions, including grief and anger, in a more authentic way. In contrast, working with interpreters presents challenges: even professionally trained language mediators may affect the intimacy of therapy, and using lay interpreters (e.g., family members) is professionally discouraged [5,6]. For these reasons, experts advocate for offering native-language psychotherapy wherever possible, or at least providing culturally sensitive language support [1,5]. In summary, according to the recent literature, the choice of therapy language is not a neutral element of the therapeutic setting but may actively shape how clients engage with emotional materials and perceive therapeutic safety [7].

### 1.3. The Person-Centered Approach as a Theoretical Framework (Empathy, Authenticity, and Language)

The person-centered approach developed by Carl Rogers offers a valuable theoretical foundation, based on the core attitudes of empathy, unconditional positive regard, and congruence (authenticity) [8]. These attitudes are intended to foster a non-threatening, trust-enhancing environment, supporting the possibility that the unfamiliar may be explored and gradually integrated [9].

Characterized by non-judgmental attentiveness, empathy, and congruence, the person-centered approach is inherently incompatible with exclusion or discrimination. In the context of refugee therapy—where experiences of discrimination and loss of trust are common—Rogers’ stance is seen as a potential secure base from which difficult experiences may be explored and expressed [9]. Empirically, it is well established that the therapist’s empathic understanding significantly contributes to therapeutic success. A recent meta-analysis has confirmed that therapeutic empathy is a moderately strong predictor of positive outcomes (average effect size r ≈ 0.28) [10]. The authenticity of therapists (congruence) is also significantly correlated with outcomes, with studies showing small to medium effect sizes (r ≈ 0.23) [11]. The person-centered perspective posits a close link between language and empathy, suggesting that a shared linguistic and metaphorical framework may support a deeper understanding of the client’s subjective experience [5,6]. The literature describes the therapist’s attunement to a client’s linguistic and cultural background as a way to express authenticity and respect for the client’s lived experience. Such attunement is assumed to support trust-building and a therapeutic encounter based on equality—principles that are central to the person-centered process [6,9]. Overall, the person-centered approach provides a useful framework for conceptualizing the roles of empathy, authenticity, and language in the context of traumatized refugees. Its core principles guide therapists in creating culturally sensitive, linguistically attuned, and empathic relationships that support healing processes.

### 1.4. Research Gap: The Role of the Mother Tongue in Refugee Therapy

Although clinical observations suggest that therapy language may play an important role, research on the specific impact of the use of mother tongue in psychotherapy with refugees remains sparse. Earlier studies on refugees mainly focused on the prevalence of PTSD and depression as well as traumatic events [2]. In contrast, the role of language and culture in the therapy process has rarely been examined in a structured manner. A recent review noted the lack of robust data on the prevalence of mental illness in diverse refugee populations and the full spectrum of psychological problems [2]. In particular, few studies have systematically examined and described therapy language as a variable, such as exploring whether the use of the mother tongue compared to a second language is associated with different therapeutic outcomes. One article even posed the “central question” of whether a fundamentally emotional therapeutic process can be effective at all if the clients’ mother tongue plays no role [7]. While recent guidelines (e.g., from the European Psychiatric Association) emphasize the importance of native-language services, they also point out the lack of research in this area [5]. The existing literature on the use of interpreters or culturally sensitive therapy offers valuable practical insights, but there is a lack of controlled studies and meta-analyses specifically addressing the effectiveness of native-language psychotherapy for refugees. This research gap has only recently been acknowledged, for instance, in case studies by Tannenbaum and Har (2020), who examined the position of the first language in cognitive–behavioral therapy with immigrants [7]. There is a broad consensus that further research is needed in this area [1,2]. Identifying this gap is of particular relevance, as it formed the starting point for the present study. The aim was to explore to what extent, and under which conditions, the use of the clients’ native language may offer therapeutic value—an issue that has not been addressed to date in a structured manner.

Based on these considerations, the following research questions guided this study:

**RQ1:**  *To what extent has the role of native language been empirically or conceptually studied in the context of psychotherapy for displaced persons?*

**RQ2:**  *What can be learned about the potential and limitations of native-language therapy from a person-centered perspective?*

### 1.5. Need for a Review

Given the ambiguities outlined above, a structured narrative review appears both necessary and appropriate. This approach allows for the collection and critical appraisal of all available studies to determine whether reliable findings already exist or whether the evidence remains inconsistent. In particular, in the field of refugee mental health, the findings are often heterogeneous and methodologically diverse, complicating synthesis [2]. Several authors have pointed to the limited reliability of existing findings: although numerous intervention studies exist, their validity is often reduced by low methodological quality [12]. One recent review found that although certain psychological interventions appear promising, confidence in this evidence is low to very low. Similarly, a Cochrane review concluded that many studies carry a high risk of bias and lack standardized outcomes [13].

A structured narrative review can address this problem by applying predefined inclusion and exclusion criteria and systematically synthesizing results in a transparent manner. Although not a systematic review in a strict sense, the present review was conducted in accordance with key PRISMA principles, including a transparent search strategy, clearly defined selection criteria, and a logical structure for data extraction and analysis. This approach allows for a critical synthesis of findings regarding the potential impact of therapy language on treatment outcomes and provides insight into whether generalizable recommendations can be drawn from the available evidence. In addition, this review helps identify research gaps (e.g., lack of studies for specific subgroups or settings). In short, conducting a structured narrative review is warranted to establish the current state of knowledge and to provide a sound foundation for answering the research question [1,2]. If the review reveals that the evidence is thin or of low quality, this would underscore the need for further primary research.

### 1.6. Thematic Analysis as a Synthesis Method in Qualitative Reviews

For this investigation, which included heterogeneous qualitative and mixed-methods studies, thematic analysis (or thematic synthesis) is a suitable method of analysis. Thematic analysis is an established method for identifying recurring patterns and key categories in qualitative data [14,15]. In the context of reviews, this method is frequently used to integrate findings from multiple studies and derive overarching insights. Thomas and Harden (2008) were the first to systematically describe how qualitative findings from different primary studies can be synthesized thematically to generate new insights [16]. Since then, this approach—sometimes in the form of framework synthesis or meta-ethnography—has become a standard in qualitative evidence synthesis. In particular, when the literature is conceptually diverse or methodologically varied, thematic analysis enables the structuring of findings at an abstract level. Individual study results are compared and grouped under common themes or concepts. For example, across various case studies and practitioner reports on therapy with refugees, themes such as “language barriers,” “cultural understanding,” or “therapist–client relationship” can be extracted. The intended thematic synthesis followed established steps: coding of the primary study findings, grouping them into initial themes, and iteratively refining them into main themes [14,15]. Through this transparent process, structured insights were generated that go beyond single studies and help to illustrate the bigger picture. This approach is widely accepted and recommended for qualitative reviews in health research [15]. It enables the integration of diverse literature—including interviews, case reports, and observational studies—into a coherent whole and facilitates the formulation of practice-relevant conclusions. Thematic analysis is particularly suited to reviews aiming not only to list findings, but to organize them conceptually—should the data allow for such synthesis. This method therefore offers an appropriate framework for exploring the potential multi-layered role of therapy language in refugee mental health based on qualitative materials [15,16].

## 2. Key Definitions in Psychotherapy Contexts

### 2.1. Migration, Forced Displacement, and Refugees

In psychotherapeutic research, migration typically refers to the voluntary movement of individuals or groups across borders, often for reasons such as work, education, or family reunification. In contrast, forced displacement—for example, of people categorized as ‘refugees’, ‘asylum seekers’, and ‘displaced persons’—denotes migration under duress due to war, persecution, or disaster. These distinctions are crucial for understanding the specific mental health needs of displaced individuals.

According to [17], forced migration is associated with unique psychological stressors such as traumatic exposure, loss of homeland, and uncertain legal status. The UNHCR [18] defines refugees as individuals who are forced to flee their country due to conflict, violence, or persecution and are unable to return safely.

### 2.2. Language in Psychotherapy

Language in psychotherapy is not merely a communicative tool but a therapeutic medium that co-constructs meaning, emotional regulation, and relational depth. Psycholinguistic attunement—where the therapist adapts to the client’s linguistic and cultural repertoire—is essential for therapeutic efficacy.

According to [19], multilingual therapists report that clients often display deeper emotional access and greater spontaneity when speaking their native language in therapy. The authors emphasized that language choice is not merely a practical matter, but deeply intertwined with emotional safety, identity, and therapeutic attunement. Language seems to operate as a carrier of emotional and cultural meaning in therapy. Recent studies have emphasized the importance of language matching between therapist and client to foster psychological safety, alliance, and disclosure [20].

### 2.3. Native Language in Psychotherapy

The existing research suggests that using a client’s native language in psychotherapy may facilitate emotional expressiveness, access to early memories, and authentic self-exploration.

Research by Dewaele and Costa [21] suggested that emotional resonance is stronger when clients communicate in their native language. Ref. [7] emphasized that native-language psychotherapy tends to be more emotionally meaningful and may enable deeper therapeutic processing.

## 3. Methodological Approach

This study was conducted as a structured narrative review that followed the methodological principles of systematic reviews as outlined by [22,23], adapted to the context of psychotherapy and the social sciences. The entire review process was guided by the PRISMA 2020 guidelines and was transparently documented and preregistered via the Open Science Framework (OSF) [24].

Data collection and study selection were based on clearly defined inclusion and exclusion criteria, with a structured screening process and presentation via a PRISMA-compliant flow diagram. The synthesis of findings was conducted through a thematic analysis, a rigorous qualitative approach suitable for identifying, analyzing, and interpreting patterns of meaning across complex datasets [25]. This method has gained increasing recognition for its ability to synthesize qualitative findings within structured review designs [16,26].

In the context of this review, thematic synthesis was used not only to categorize existing evidence but to generate deeper conceptual insights into how native-language psychotherapy is understood and practiced in refugee settings—particularly within a person-centered therapeutic framework. This integration of systematic identification, transparent documentation, and qualitative interpretation reflects an emerging best practice in psychotherapy research and enables a nuanced understanding of the role of native language in therapeutic processes.

### 3.1. Criteria

In terms of language, this structured narrative literature review was based on both German- and English-language publications.

The combination of information obtained from the EBSCO databases, particularly PSYNDEX, PsychOpen, Psychdata, PubPsych, and additionally from the PCE Literature database, which specializes in person-centered literature, as well as the review of all the issues of the two central journals (PERSON and Person-Centered and Experiential Psychotherapies (PCEP)) provide a strong indication of the status quo and served as a representative basis for the present topic.

In the course of the research, however, it turned out that there was not enough material on this topic in peer-reviewed journals, so the criteria were expanded to include scientific reports, books, analyses, and other types of scientific literature.

No delimitation was necessary with regard to the year of publication, especially as it became apparent in the course of the research that the total number of publications was low.

In terms of content, publications involving the importance of native-language psychotherapy for traumatized (war) refugees were considered.

### 3.2. Search Factors and Implementation

The only restriction was that the search terms had to be found primarily in the title, abstract, or keywords. In cases of uncertainty, a full-text search was also carried out. The use of synonyms of the main search terms significantly changed the number of hits during the search.

There were only a few German- and/or English-language publications that addressed the topic of psychotherapy conducted in the native language of immigrants/displaced and traumatized people without restriction. For this reason, all the articles included in this review only partially provided a basis for explaining this topic.

In the next step, the search of the German-language literature was expanded to include “intercultural psychotherapy”. This yielded 64 publications, of which only 14 were published in peer-reviewed journals. The search for “psychotherapy with interpreters”, which is a completely different aspect, yielded 21 hits, 9 of which were peer-reviewed. “Psychotherapy with refugees” or “asylum seekers or displaced persons” produced a total of 190 publications, over half of which were peer-reviewed. After closer examination and narrowing down of the results, it was found that the aspect of native-language psychotherapeutic treatment was not studied in detail in most of the retrieved articles.

The author only found 18 German-language publications (7 peer-reviewed), with the most important terms mostly found in the title and keywords. As soon as a further link to (war) flight and trauma (including synonyms) was made, it reduced the number of publications to five, of which only two were peer-reviewed.

Changing the language to English resulted in almost two thousand hits for “psychotherapy with refugees/asylum seekers”. When the search was refined using the terms “native language/mother tongue/first language” and “trauma”, there were again only a few results: six publications (with only one peer-reviewed publication).

It can be seen that scientific discussions in this area are developing primarily around multilingualism and interculturality in psychotherapy in general, although this is a different target group. Migration, which is usually consciously chosen and experienced—including the deliberate choice to learn the new language—cannot be compared with an often unprepared (war) flight scenario with all its consequences, such as not speaking the foreign language. The use of interpreters, including the associated advantages and disadvantages, is also frequently discussed. However, very few publications were found that explicitly addressed the importance of native language skills (for therapists and clients), including their consequences in psychotherapy for this target group. There also seems to be sufficient research into the factors that are considered in migrant psychotherapy, but not into the psychotherapeutic treatment of war-traumatized displaced persons/refugees/people seeking protection and the resulting issue of psychotherapeutic support in the (native) language of these people.

An attempt to focus the search on purely person-centered psychotherapeutic literature (PCE Literature database, all issues of PERSON and PCEP) did not expand the results.

Finally, a combined search of all the sources mentioned, in both languages and using additional specialized libraries, conferences and, above all, targeted letters to the main authors in this field, yielded a list of 33 publicly accessible scientific publications that were analyzed thematically. Twelve of these were subjected to an additional, more detailed thematic analysis.

### 3.3. Reflexivity and Researcher Positioning

The author acknowledges their background as a psychotherapist and trauma researcher with professional experience in working with displaced individuals. This background has influenced the formulation of the research question and the interpretative lens applied during the thematic synthesis. To reduce potential bias, a reflexive approach was adopted throughout the research process. This included maintaining a reflective log and revisiting key coding decisions to ensure alignment with the data rather than with preconceived assumptions. The author remained attentive to the possibility of overemphasizing familiar concepts or therapeutic ideals and actively sought alternative explanations during the analysis phase.

### 3.4. Language and Technology Considerations

All screening and data extraction steps were conducted manually without the use of artificial intelligence tools or automated processes. The decision to rely exclusively on human screening reflects both the complexity of language-specific nuances in the psychotherapy literature and a commitment to a careful, context-sensitive interpretation of each source. The author acknowledges that such an approach is time-intensive but considered it essential for ensuring the methodological integrity of the qualitative review process.

## 4. Results

### 4.1. Cluster 1—Conceptual Grounding

#### 4.1.1. Origin of the Problem—Flight and Migration as a Crisis

Due to political upheaval, social upheaval, and climate change, migration is becoming a global phenomenon. A subgroup of migrants are refugees, i.e., people who have left their home country involuntarily due to war, natural disasters, religious or political persecution, etc., in order to seek protection elsewhere [27]. A current example of this is Ukrainian displaced persons who, due to the sudden outbreak of war in Russia against Ukraine, are seeking a place to stay and have to establish a new center of life in a country that they previously had no linguistic or cultural connection to. Refugees have some things in common with other groups of immigrants, especially with regard to their experiences after migration, such as various experiences of loss (e.g., social networks) and overcoming communication barriers by learning a new language. However, they differ greatly, especially with regard to their experiences before and during migration [28].

Although many psychotherapists have experienced migration themselves, culture-specific and intercultural aspects only found their way into the psychotherapeutic literature very recently. One exception is the monograph of [29]. Here, migration is understood as trauma or a life crisis. Grinberg and Grinberg [29] referred to the model of [30], which describes the psychological development of migration in three stages. In the first phase, the differences between the new objects and the psychological representation of the abandoned culture become clear; in the second phase, the individual becomes free to appropriate the new objects through mourning; and, finally, in the third phase, the individual develops a new self-concept [29].

However, the specialist literature clearly shows that conscious, voluntary migration with appropriate preparation (pre-migration) has a high probability of not leading to a personal crisis and, therefore, does not automatically cause mental illness and its consequences. The situation is different in the case of flight or flight-like experiences. In her numerous publications on this topic, Erim states that “involuntary” migrants (refugees, displaced persons, and asylum seekers) are more stressed than “voluntary” migrants (e.g., migrant workers). In a review that included almost 25,000 migrants from 18 countries, Lindert et al. [31] also found that the prevalence rates of depression, anxiety, and post-traumatic stress disorder (PTSD) among refugees were twice as high as among those who migrated for other reasons) [28].

The term “multicultural counseling” as “psychology’s fourth force” was also not mentioned in person-centered literature until the early 1990s. From a historical perspective, this can be explained by the fact that the person-centered approach was originally presented as a form of therapy that was equally suitable for all clients. Since 2000, in particular, many publications on the need for differentiation and on culturally sensitive person-centered psychotherapy and the importance of transcultural competence have appeared [32].

While the importance of (cultural) diversity in the person-centered therapeutic process has since been increasingly addressed by researchers, the importance of communicating in the same language has received little attention and requires further consideration from a person-centered perspective.

#### 4.1.2. Disorders as a Consequence—“Only” Trauma or More

According to the literature examined, trauma sequelae are among the most common psychological diagnoses in the treatment of refugees, displaced persons, and people seeking protection. However, there are also studies [33] that examined somatoform disorders, depressive disorders, and addiction tendencies in this context.

The available studies show that mental health problems in immigrants are often recognized too late in primary care. For example, Odell et al. [34] studied patients in general practices in West Birmingham, and found that mental health problems were less likely to be recognized in patients of Asian descent and African-American patients than in other population groups. Mental health problems were more likely to be recognized in immigrants if they were related to social problems or if the patients had a psychiatric history. Concomitant somatic illnesses, on the other hand, hindered the recognition of mental health problems. Even in the case of obvious behavioral problems, diagnosis can be complicated by language and cultural communication problems. In their empirical study, Haasen et al. [35] were able to demonstrate the difficulties in making a psychiatric diagnosis when the patient has insufficient language skills.

Gavranidou [36] also mentioned several triggers that influence the possible development of mental illness in connection with emigrants or refugees. These can be migration-related factors (arising through/from migration as a process, such as language problems, legal status, and culture shock), but also factors unrelated to migration (divorce, unemployment, loss of a child, etc.) as well as culture-specific factors (educational ideas and expectations of the group towards the individual) or trauma-related factors (traumatic events in the home country, during flight, and in the host country).

According to [28], migrants and refugees also suffer from dual needs [37]: psychological and social problems that arise in their host country after fleeing and thus promote the development of psychological consequences. They are often affected by unemployment, residence restrictions, class-specific problems, different levels of education (both among native and various subgroups of immigrants), low participation in prosperity, discrimination at school and in administrative aspects, and when looking for work [28].

To ensure that these psychological conditions are not overlooked and to avoid re-traumatization, chronification, transgenerational transmission of trauma sequelae, and other mental illnesses in this vulnerable target group as a consequence of refugee-like experiences, psychotherapy in the mother tongue is recommended [28], including cultural diagnostics/anamnesis, if foreign language skills are insufficient and in the case of particularly pronounced traumatization. DSM-5 recommends the Cultural Formulation Interview (CFI) for this purpose [38]. Among other things, the CFI covers the patient’s cultural identity, language skills, value orientations, illness concepts, and self and body images.

An important contribution [9] to answering the question of person-centered resources for psychotherapeutic work with clients with a migration history examined, among other things, the areas of tension arising from migration and reported that a person-centered approach can, by its very nature, create a non-threatening, trustworthy framework for encounters, whereby the unfamiliar can be better understood and integrated by recognizing, naming, and acknowledging differences. Stressful experiences can be better symbolized and dealt with constructively if a verbal challenge can be overcome and a common language can be found.

The following cluster provides essential contextual groundwork for understanding the psychological burden and therapeutic needs of displaced individuals. Although not addressing the research questions directly, this thematic section frames the relevance of language in therapy by illustrating how forced migration, trauma exposure, and diagnostic barriers shape the mental health landscape in which psychotherapy—particularly in the client’s mother tongue—unfolds.

### 4.2. Cluster 2—Language Use and Access to Psychotherapy

The following cluster gathers studies that examine how language use shapes access to psychotherapy for refugees and displaced persons. It highlights how the absence of a shared therapeutic language can impede diagnosis, trust, and therapeutic engagement, while native-language use is associated with deeper emotional processing and reductions in miscommunication. These findings directly contribute to answering RQ1, which addresses how native-language psychotherapy is applied in practice and where access barriers emerge.

“It’s called mother tongue for a reason. They are words that have a very deep emotional meaning and impact that just can’t exist in a second language without them being a little thinner in meaning. This is the language you were raised on. It was what you were sung to as an infant. It was the words that we love. The endearment … or terror”

This quote from the qualitative study on bilingual psychotherapy from the therapist’s perspective by [39] (p. 104), which highlights the importance of the mother tongue in general and from the perspective of psychotherapeutic work with foreign clients in particular. The mother tongue appears to be of central importance for the organization of experience, emotion, and memory as well as being of particular importance in the treatment of trauma and its consequences.

The authors [39] go on to say that bilingual clients (if they speak at least one foreign language in addition to their mother tongue) tend to fall back on their mother tongue when expressing strong emotions or when dealing with death or, in particular, trauma or when dreaming. They may also use their first language to protect themselves from painful experiences. The respondents in the study by Kokaliari et al. [39] agreed that language is a factor that influences the therapeutic alliance, as issues of trust, idealization, and hostility towards the psychotherapist are encoded in language. The original definition of language by Stern defines the mother tongue as the crucial, primary basis for emotional development and intersubjective understanding [40]. Encoded in language are the physical tone of the early caregiving environment, the cadence and rhythm exchanged between the caregiver and the child that soothe, excite, frighten, and shape the meaning of the child’s world [40,41,42,43]. Feelings such as love, anger, frustration, security, fear, and self-awareness are associated with the caregiver’s words and encoded as aspects of the earliest experiences [44,45]. Language also functions at the level of associations and meanings, which are rarely translatable [42,46,47]. If a person’s experiences are transferred from the primary language in which they were made to a new language, fewer details are often translated, and the vividness is reduced or lacking [43]. Therefore, people may have significant discrepancies in their experiences from language to language [48]. Traumatic experiences, such as those during flight, have a special position in communication. The more stressful and traumatic an experience was, the more likely it is that it will be processed and expressed in the language of the context in which it was made [47]. The ability to learn to deal with traumatic experiences in their mother tongue can help clients to process repressed memories and to work through events and details [43,47,49] in a way that would not be possible in a second language. However, in cases where therapists are not bilingual, there may be negative consequences. Some clients may be overwhelmed if too much traumatic material (experiences, emotions, and memories) is expressed in their mother tongue without the therapist knowing what is being said, experienced, and acted out. Therefore, speaking in the mother tongue can act as a trigger and also initiate a regressive experience that can overwhelm the client if it is not accessed and/or controlled by the therapist [50].

There is also evidence in person-centered research of the impact and importance of language in therapy situations. Lux [51], for example, specifically looked at the impact factors of person-centered relationship-building, such as empathic understanding from a neuroscientific perspective, and found that psychophysiological processes appear to synchronize in the people involved in intense moments of an encounter. This phenomenon is known as neuronal coupling. It was shown that when listening to a story, the activation patterns in many areas of the brain synchronized with each other. However, as expected, this was not the case when the story was presented in a language that was incomprehensible to the participants [51].

So what are the indications that native-language psychotherapy might be necessary? First and foremost, there are obvious communication problems that make it clear that treatment should be carried out in another language in order to avoid misdiagnosis and the resulting incorrect treatment. Taking into account the ambivalent relationship between diagnostics and person-centered psychotherapy, which rejects status diagnostics, special importance is attached to processual diagnostics in the course of therapy [52]. However, processual diagnostics also primarily require verbal communication.

Mother tongue psychotherapy is therefore always useful when the patient’s and/or therapist’s knowledge is not sufficient to express themselves emotionally and intellectually in the same language (usually that of the host country). In combination with intercultural competence, native-language psychotherapy enables therapists to make a more reliable assessment in many cases regarding, for example, whether certain linguistic expressions (e.g., “my internal organs are burning”—a melancholic delusion or culturally influenced use of language?) or a certain behavior (patient does not take medication and is being treated by a religious healer—delusion or culturally influenced attempt at healing?) indicate a cultural attitude or a mental disorder [53].

According to [53], people suffering from post-traumatic stress disorder (PTSD) are particularly at risk of reduced effectiveness of therapy if it is not adapted to their native language.

Taken together, these findings synthesize both the empirical and conceptual contributions that illustrate how the use of native language is addressed in current refugee psychotherapy practice, providing an answer to RQ1.

### 4.3. Cluster 3—Native-Language Therapy as Mechanism of Change

The studies grouped in this cluster emphasize how psychotherapy in the client’s native language can serve as an active therapeutic mechanism. Rather than being seen as a deficit, the use of the mother tongue is portrayed as a resource that fosters emotional depth, trust, motivation, and congruence. These findings provide insight into person-centered mechanisms of change—such as empathy, authenticity, and acceptance—that are strengthened when communication occurs in the client’s native language. As such, this cluster directly addresses RQ2, which explores how native-language therapy supports key therapeutic processes from a person-centered perspective.

“Less drop out, more compliance and motivation, trust, deeper dynamics, no taboos” is a fitting headline to summarize the overwhelming advantages of mother tongue psychotherapy as a resource from the author’s point of view.

However, if this is considered in detail and analyzed step by step, the term “mother tongue treatment (in therapy)”, according to [54], is mainly found in the German-language literature published in the 1990s [55,56,57]. In the international specialist literature, two comparable and matching terms emerged a little later: Kirmayer et al. [58] defined “cultural consultation service” and Tantam [59] described “ethnic matching” as the treatment of migrants by therapists from the same ethnic group and thus communicating in the same language. Tantam [59] further argued that therapists are more likely or quicker to develop empathy for patients of the same ethnicity when empathy is understood as an emotional contagion nurtured by previous (similarly experienced) relationships, values, and expectations. Relevant arguments in favor of native-speaking or ethnically appropriate psychotherapists are that their use leads to a higher utilization of facilities by migrants, as well as better therapy motivation and compliance.

However, critical voices can also be found in publications by person-centered researchers. Ethnic matching is described as a complex phenomenon and attention is drawn to the considerable organizational challenges involved in managing and predicting the success of such working relationships [32].

According to [54], a particularly conducive prerequisite for intercultural psychotherapy is a native-language service provided by bilingual psychotherapists; for example, in an in-patient care facility [60,61,62,63]. In this context, bilingual “native-speaking” therapists can make the team understand which cultural characteristics may have played a role in the development of the symptoms. This model makes the recognition of other cultural affiliations visible, which can be enriching for everyone involved.

Another well-known way of circumventing the language barrier is to use interpreters. At the same time, many publications draw attention to the limitations of this method, unless they are specially trained [64]. For example, a possible—and not infrequent—occurrence is self-interpretation of the therapy content (e.g., due to divergent cultural values, religious views, or gender issues). In addition, interpreters have often not undergone psychotherapy training and are therefore less able to differentiate themselves from content that is difficult to digest. Toker [65], for example, emphasized the challenges of using interpreters in psychotherapy and advocated referring foreign patients to native-speaking psychotherapists whenever possible in the event of culture-specific or communication problems. Baxter and Cheng [66] also reported having used bilingual supervision after their own experience with interpreter-assisted psychotherapy. However, the use of interpreters is recommended for brief and acute crisis interventions or diagnostic consultations. In her publication on the framework conditions of triadic psychotherapy, Pinzker (2018) [64] drew attention to the lack of research focus, particularly in the person-centered specialist literature, and set out to find ways to facilitate person-centered approaches in the daily work of the triad and called for further in-depth studies from a person-centered perspective.

As therapists from different schools and approaches discuss the importance of mother tongue psychotherapy and critically reflect on the use of their own methods (joining, re-framing, biography work, fairy tale therapy, etc.) in this context, the basic features of person-centered psychotherapy and Rogers’ attitude dimensions are increasingly being seen as a very good starting point. Some authors have argued that Rogers’ universally valid attitude dimensions should be supplemented by the goals, motives, and needs of the migration process and cultural affiliation in person-centered relationship design [36]. Erim [67] sees similarities between mother tongue treatment and the principle of “peer counseling” according to Rogers, which emerged in the context of the “self-determined living” movement of people with disabilities in the USA [68]. It is based on the experience of self-help groups in which people in a similarly difficult life situation support each other and exchange ideas. This leads to a more conscious experience of one’s own history and identity.

Resource orientation or activation of ethnic resources (personal, familial, social, and bicultural) instead of deficit orientation is another promising perspective in mother tongue psychotherapy, although other views were initially widespread in the history of interethnic psychotherapy. Tang and Gardner [69] as well as Holmes [70], for example, described that until the 1960s in the USA, among psychotherapy patients, differences observed in an ethnic group were regarded as a deficit of the ethnic group and as a treatment problem. We now know, from the widely cited literature, that migration should be regarded as an important life event that is stressful for some but does not necessarily have to lead to psychological problems, given that it—as previously mentioned—can also lead to enrichment, in that it opens up new possibilities for action for the individual and can lead to a new self-concept [29].

From a person-centered perspective, a person’s self-concept is subject to constant change through every new experience, such as migration [52]. A developmental disorder occurs when a person’s evaluations of an experience (e.g., negative experiences in a new country) deviate from the evaluations of the self-concept and a defensive reaction occurs. Growth with the help of person-centered psychotherapy is possible through cognitive restructuring of the evaluations experienced as feelings. The self-concept is experienced in greater congruence with the organismic experience, which enables development.

The basic attitudes of person-centered talk therapy have a supportive effect in the therapeutic accompaniment of a migration process, but verbal communication remains a prerequisite.

### 4.4. Cluster 4—Risks, Boundaries, and Therapeutic Fit

While the benefits of native-language psychotherapy are well documented, this cluster addresses its potential risks and limitations. The included studies illustrate how language—though often a therapeutic asset—can also present challenges related to cultural projections, transference phenomena, and linguistic nuances. These aspects require careful reflection in therapeutic practice, especially in person-centered settings where congruence and mutual understanding are central. Cluster 4 thus extends the answer to RQ2 by revealing both enabling and complicating factors in the use of the mother tongue as a medium for person-centered therapy.

As much as most experts advocate the use of native-language psychotherapy—both for traumatized refugees/displaced persons and for immigrants of another ethnicity who do not speak another foreign language sufficiently to undergo therapy in this new language—there are also hidden dangers and aspects to be considered.

For example, although successfully integrated therapists from the same ethnic group are reported to be primarily seen as role models or even saviors by clients, there are also opposing views and the risk of being identified as enemies, which can be counterproductive for relationship building and the potential therapy process. For example, some colleagues report that cultural similarities also trigger feelings of envy when clients perceive the therapist’s success as a betrayal of the original culture [71].

Furthermore, some studies point out that interethnic therapists—especially those from the second or third generation of migrants—may not always have sufficient command of their heritage language to address all, and particularly emotionally complex, therapy topics. This challenge is especially relevant in therapeutic work with refugees or displaced persons, where therapy in the mother tongue is explicitly requested or assumed. Although these therapists often speak the language fluently in family or informal contexts, their professional training and education have typically taken place in the majority language of the host country. As a result, gaps in therapeutic vocabulary or clinical fluency may occur, and important topics might be avoided, sometimes due to uncertainty or shame [56].

In addition, some studies mention linguistic nuances such as dialects, slang, and accents, which can complicate mutual understanding even when the therapist and client technically speak the same language. This is particularly relevant in therapeutic settings involving refugees or displaced persons from multilingual regions. Caution is also advised when a shared language is assumed but turns out to be a second state language in the client’s country of origin—one that may carry historical or political baggage. Examples include Russian in Ukraine or Chechnya, or Turkish among Kurdish minorities. This is often the case when the first language is spoken by a minority whose language is not popular or known internationally and there are hardly any therapists or interpreters who speak it. If there is no other foreign language to choose from, the common second language is used, which was/is in use in several countries at the same time. This approach facilitates therapy and, ideally, has a similar effect to treatment in the mother tongue, but requires a far more sensitive approach and empathetic consultation such that there are no (unspoken) obstacles or even hostility [39,72].

As already mentioned, caution is also required with some mental disorders. On the one hand, psychotherapy in the mother tongue may help to address trauma sequelae or facilitate the discussion of difficult topics—particularly in the context of forced migration. Nevertheless, some studies suggest that, for individuals experiencing psychotic symptoms—whether as pre-existing conditions or as part of post-migration mental health challenges—avoiding the mother tongue might be beneficial to reduce symptom intensity or psychological distress [39].

The cultural values of the original culture and language must also be taken into account, especially in refugee contexts where sociocultural taboos may persist despite displacement. For example, some displaced persons or immigrants avoid using their mother tongue in therapy because it may limit the expression of internal conflicts or socially forbidden aspects of the self. Cultural prohibitions regarding sexuality or aggression can provoke shame or guilt when described in the native language, leading to the preference for a second, more emotionally neutral language. Therefore, there may be a desire to switch to another language that is less affectively charged and more permissive. The second language, if available and sufficiently mastered, can then provide a space for healing [39,44].

Similarly, a second language can enable new kinds of experiences, e.g., a new way of expressing ethics or even power dynamics that may not have been allowed in the main language [73,74].

From a psychoanalytic point of view, the reviewed literature emphasized the potential dynamics of transference and countertransference [71], primarily in bilingual therapeutic settings. Although not exclusive to refugee contexts, these dynamics may be intensified when therapist and client share linguistic or cultural backgrounds. The risk of over-involvement (over-joining) or emotional distancing—especially when cultural similarity is presumed—may complicate the therapeutic relationship and the capacity to address core issues [39].

While the reviewed literature does not explicitly offer a person-centered interpretation of the challenges outlined above, some implications may be cautiously considered. Although the person-centered approach does not typically conceptualize transference and countertransference in the classical sense [52], therapeutic tensions can still arise—particularly when working in the client’s mother tongue. In such situations, a person-centered focus on congruence, empathic understanding, and dialog in the here and now may help to clarify misunderstandings, address emerging discomforts early, and support a balanced therapeutic relationship.

Provided that psychotherapy in the mother tongue is given more attention as a resource in the future and that the psychological consequences of traumatization during (war) flight, displacement, and persecution can be more easily absorbed and treated in a timely manner, chronification and comorbidities can be avoided or reduced. In this case, this would be a good step towards integration into the new country or re-integration in the event of a return home.

Taken together, the findings in this cluster offer a nuanced answer to RQ2. They highlight how person-centered rationales such as resource orientation, congruence, and empathic understanding are mediated through language. Moreover, they illustrate how mother tongue therapy can enable or challenge therapeutic processes depending on the sociocultural dynamics and client histories, thus contributing to a deeper understanding of language as a mechanism of change in the person-centered treatment of trauma and displacement.

## 5. Conclusions and Recommendations for Practice

This review confirmed that native-language psychotherapy has emerged as a critical response to the complex needs of displaced persons, particularly those who are traumatized and face communication barriers. From both a theoretical and practical standpoint, treatment in the mother tongue has proven to be more than just a tool for improved diagnostics or compliance. It functions as a relational and symbolic space, where clients can begin to process and integrate traumatic experiences into their evolving life narratives.

As emphasized by [75], native-language therapy does not serve to idealize one’s culture of origin or isolate clients from the host society. Instead, it can open up a “transcultural development space,” enabling exploration and negotiation between cultural values in a psychologically safe environment [67]. To accompany clients through this process, transcultural competence and a capacity for critical self-reflection on the part of therapists are essential. This includes recognizing pseudo-empathy and cultural arrogance [36], as well as examining one’s own positioning within asymmetric power structures.

The analysis also raises important practical questions: What alternative options are available when there are too few multilingual therapists? How can psychological healing still occur in such settings? The literature offers promising alternatives, including group therapy formats, which have proven effective in collectivist cultures and are often better received when they are gender-specific [76]. In addition, online psychotherapy by therapists from the clients’ country of origin has shown promise, especially in mitigating geographical access issues [77]. Nevertheless, this raises unresolved legal and regulatory questions about licensing foreign-trained therapists in host countries—a task for policymakers and relevant professional bodies.

Supporting materials also play an essential role; these include culturally sensitive diagnostic tools, intercultural practice guidelines, and the use of tools such as the Cultural Formulation Interview (CFI), as presented in the DSM-5 [78], which can significantly enhance diagnostic accuracy and therapeutic alliance. These tools help to assess not only linguistic fluency but also cultural identity, value systems, illness beliefs, and the client’s experience of suffering—all of which are critical to the success of any psychotherapeutic process.

From a person-centered perspective, the therapeutic process relies heavily on the symbolic power of language to help clients give meaning to their lived experiences. While it is acknowledged that empathy and unconditional positive regard can also be conveyed nonverbally, initial verbal communication remains essential—particularly when working with trauma. Native-language communication becomes a gateway to realizing Rogers’ core conditions of empathy, congruence, and unconditional positive regard. In this way, native-language therapy not only enables access but also deep therapeutic transformation.

Taken together, the four thematic clusters developed in this review respond directly to the guiding research questions. Cluster 1 contextualizes mental health needs among displaced persons and underscores the structural and societal framework of trauma and migration. Cluster 2 addresses RQ1 by illuminating how language functions as a gatekeeper to access. Cluster 3 responds to RQ2 by focusing on the change-enabling power of native-language therapy. Cluster 4 expands this analysis by highlighting possible risks, boundaries, and dilemmas arising from language use, therapeutic fit, and cultural dynamics.

The resulting insight is that native-language psychotherapy is not only a tool of access, but a core element in shaping the processes and outcomes of care. This multidimensional role of language must be recognized more explicitly in theory, training, and clinical practice.

## 6. Implications for Further Research

These findings not only carry practical implications but also reveal specific research gaps that demand empirical investigation. Building on this review, future research should aim to investigate both access-related and processual aspects of native-language psychotherapy more deeply.

Cluster 1 underscores the broader psychosocial context, migration biographies, and trauma-specific needs, which must be more systematically integrated into research designs. Cluster 2 suggests the need for empirical studies that focus on language-related access barriers, including the role of interpreters and linguistic matching. Cluster 3 calls for exploration of change processes enabled through native-language therapy, especially from a person-centered perspective. Cluster 4 urges further inquiry into risks such as emotional overload, transference, over-identification, or therapist–client mismatches.

From a theoretical perspective, native-language therapy appears to facilitate the emergence of congruence and empathic resonance, particularly when trauma narratives are symbolically integrated into the client’s self-concept. To examine these phenomena, qualitative methods such as in-depth interviews and focus groups should be used to analyze how Rogers’ core conditions are experienced or challenged in multilingual settings. This includes person-centered therapists working in native-language therapy, with interpreters, or using shared foreign languages.

In Austria, as an example, future research could build on the infrastructure of the Network for Intercultural Psychotherapy [79], which coordinates refugee-centered psychotherapeutic services. Future studies should include the following groups:

- Multilingual therapists working directly in the clients’ mother tongues;
- Therapists using interpreters or shared foreign languages;
- Refugee clients who have received various forms of therapy or no treatment;
- Interpreters with and without training in trauma-informed psychotherapy;
- Person-centered therapists with relevant clinical experience.

Based on such insights, a transnational, multilingual longitudinal study could be designed to assess the comparative effectiveness of native-language therapy versus interpreter-mediated or foreign-language psychotherapy. Sampling could include countries with high numbers of protection seekers.

In summary, future research should aim not only to close systemic access gaps, but also to understand how language supports or hinders the core processes of psychological healing. This knowledge can guide training, clinical practice, and health policy to respond more effectively to the mental health needs of forcibly displaced populations in times of crisis.

## Data Availability

Data is available upon request.
